# Antifibrotic therapy for Lower Urinary Tract Symptoms secondary to Benign Prostatic Hyperplasia

**DOI:** 10.1038/s41391-026-01119-2

**Published:** 2026-05-22

**Authors:** Ajinkya R. Limkar, Kevin T. McVary, William A. Ricke, Matthew S. Lee

**Affiliations:** 1 The University of Wisconsin, School of Medicine, Madison, WI, USA.; 2 Loyola University, Department of Urology, Maywood, IL, USA.; 3 The University of Wisconsin, Department of Urology, George M. O’Brien Center, Madison, WI, USA.; 4 The Ohio State University Wexner Medical Center, Department of Urology, Columbus, OH, USA.

## Abstract

**BACKGROUND:**

Lower Urinary Tract Symptoms secondary to Benign Prostatic Hyperplasia (LUTS/BPH) are a ubiquitous condition estimated to affect over 50% of men over the age of 50 years. Various treatment options exist for men presenting with LUTS/BPH, including lifestyle and behavioral modification and medical or surgical treatment. Although multiple medical treatments exist, innovation for the medical treatment of BPH has not significantly progressed.

**METHODS:**

A comprehensive narrative review was performed to evaluate prostate fibrosis as a contributor to LUTS/BPH and to identify potential antifibrotic therapies that may be applicable to the treatment of LUTS/BPH. A literature search was conducted in PubMed and ClinicalTrials.gov for molecular, translational, and clinical studies relevant to fibrosis in benign prostatic diseases. Keyword searches included “benign prostatic hyperplasia”, “lower urinary tract symptoms”, “prostate fibrosis”, “fibrosis”, “antifibrotics”, as well as combinations of these terms.

**RESULTS:**

In this narrative review, we review the rationale and evidence for pursuing antifibrotic therapy as a novel treatment strategy for LUTS/BPH. We discuss potential antifibrotics that could be used for the treatment of LUTS/BPH. Finally, we discuss diagnostic studies that could be used in the future to identify patients with LUTS secondary to prostate fibrosis.

**CONCLUSIONS:**

Emerging evidence suggests that prostate fibrosis contributes to the development of LUTS. No antifibrotic therapies are currently being used, leaving LUTS secondary to fibrosis untreated. Antifibrotics are a novel medication class that could be used to treat patients who progress on current BPH medications.

## INTRODUCTION

Lower Urinary Tract Symptoms secondary to Benign Prostatic Hyperplasia (LUTS/BPH) are a ubiquitous condition estimated to affect over 50% of men over the age of 50 years [1]. LUTS/BPH include various symptoms such as urinary frequency, urgency, nocturia, straining, weak urinary stream, and sensation of incomplete emptying [2]. LUTS/BPH negatively impact a man’s quality of life (QoL) [3] and are a progressive condition that, if left untreated, can have severe consequences such as detrusor deterioration, acute or chronic urinary retention, recurrent urinary tract infection, renal failure [4], and even death [5]. Various treatment options exist for men presenting with LUTS/BPH, including lifestyle and behavioral modification and medical or surgical treatment [1]. For this review, we will briefly discuss current medical BPH treatments, the scientific and clinical evidence supporting the use of antifibrotics as a novel treatment strategy for LUTS, potential antifibrotics that could be used for the treatment of LUTS, and finally, potential diagnostic tools to identify prostate fibrosis.

## PATHOPHYSIOLOGY OF LUTS/BPH

The pathophysiology of LUTS is complex and multifactorial. BPH, by definition, is a histologic diagnosis where an increased number of epithelial and stromal cells are found in the periurethral area of the prostate [6]. It is hypothesized that rather than an increase in cell proliferation, there may be a decrease in cell death leading to loss of homeostasis within the prostate and an overall increase in cellular hyperplasia [7, 8]. Multiple hormones and growth factors have been implicated in the loss of tissue homeostasis leading to prostate growth, including: androgens, estrogens, FGF [9], KGF [10], TGF-β [11], and EGF. Prostatic hyperplasia leads to increased urethral resistance secondary to physical obstruction from epithelial and stromal proliferation, which causes compensatory bladder changes and LUTS.

Counterintuitively, prostate volume does not always correlate with the degree of obstruction [12], which indicates that other factors contribute to LUTS. These factors include dynamic urethral resistance from passive and active prostatic smooth muscle dysfunction, and increased collagen deposition from prostate fibrosis. Indeed, emerging evidence points to age-mediated fibrotic remodeling, particularly within the prostate transition zone and periurethral space, as a critical mediator of the development and progression of LUTS [13–15].

**Fig. 1:**
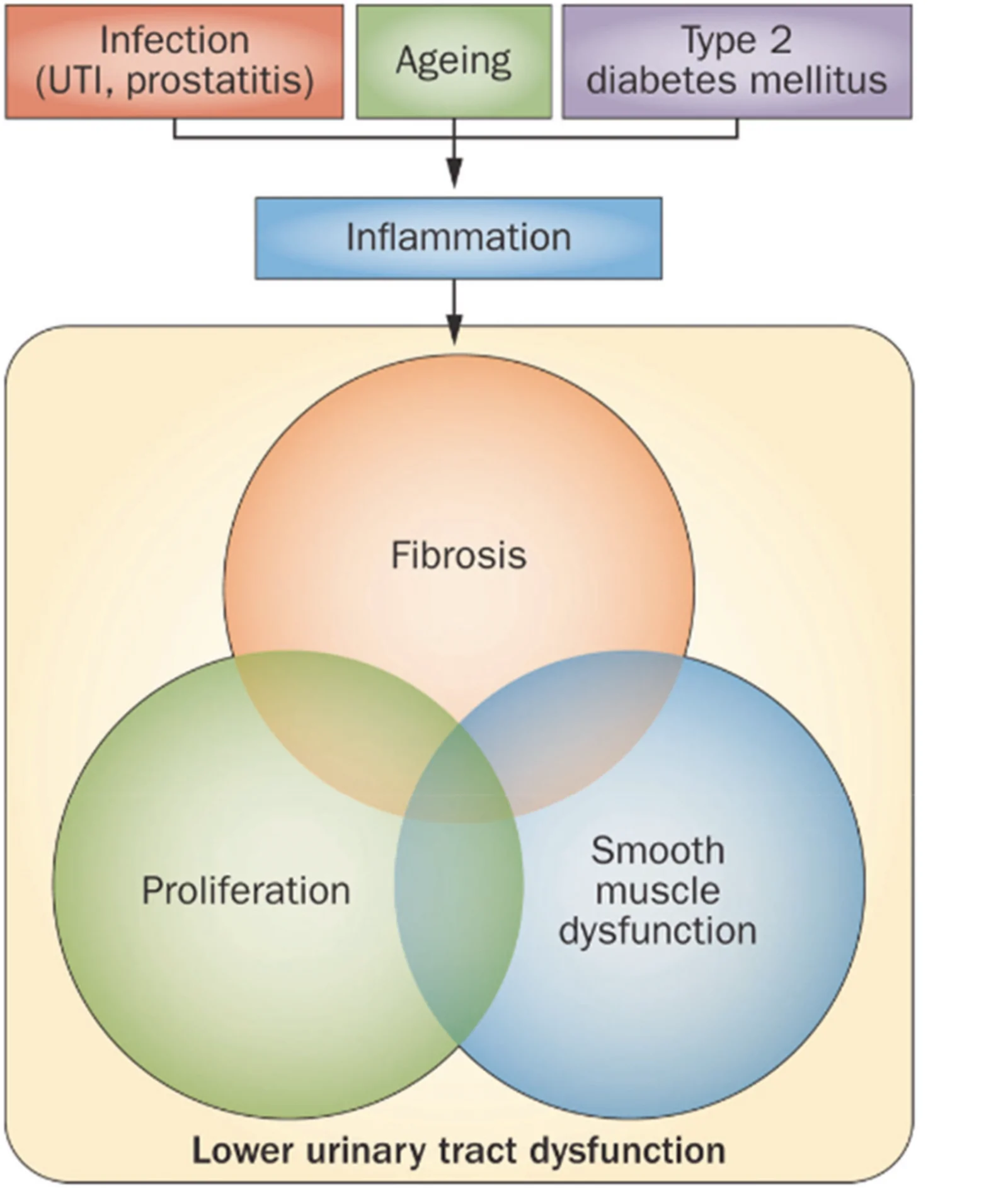
Contribution of inflammation and fibrosis to lower urinary tract dysfunction. Taken from Rodriguez-Nieves, J., Macoska, J. Prostatic fibrosis, lower urinary tract symptoms, and BPH. Nat Rev Urol **10**, 546–550 (2013) with permission.

Current medical treatments for LUTS/BPH only target prostate hyperplasia (5-alpha reductase inhibitors (5-ARI)) and prostatic smooth muscle dysfunction (alpha-blockers). Although combination therapy (5-ARI with an alpha blocker) has been shown to be more effective than monotherapy, 12.6% and 5% of men in the combination therapy arm had BPH clinical progression in CombAT and MTOPS, respectively [16, 17]. This suggests a need for additional strategies in medical BPH treatment, such as prostate fibrosis, which may be an untreated putative pathologic mechanism (Fig. 1). We will discuss emerging evidence that supports the pathologic role of prostate fibrosis in LUTS below.

## RATIONALE FOR USING ANTIFIBROTICS TO TREAT LUTS/BPH

**Fig. 2:**
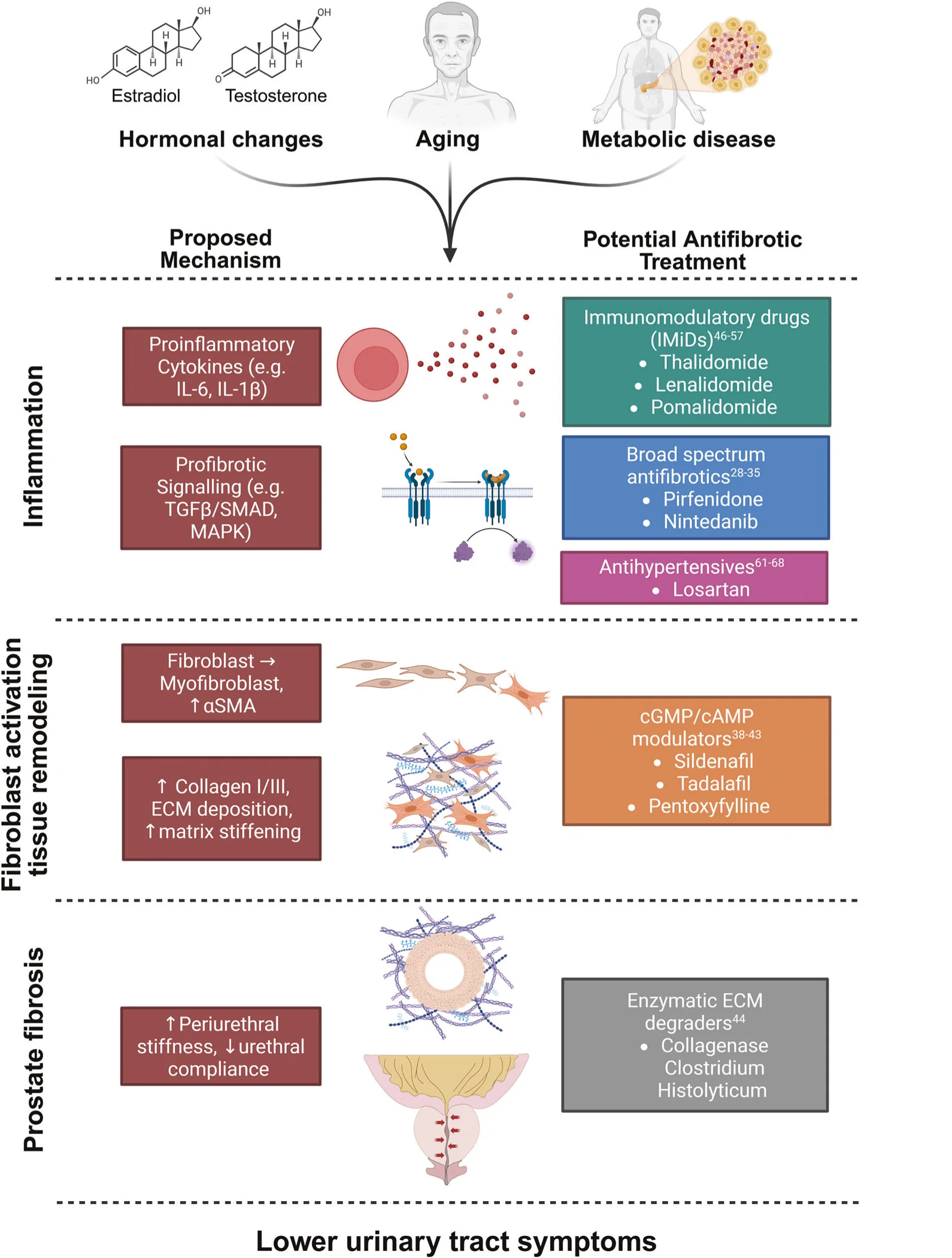
Pathogenesis of prostate fibrosis with potential antifibrotic treatment examples and their proposed mechanisms.

Tissue fibrosis is an active and highly regulated biological process that is characterized by the accumulation of extracellular matrix proteins such as collagen type I and III, increased stromal stiffness, and loss of tissue compliance. Mechanistically, fibrotic remodeling is classically driven by chronic inflammation and abnormal activation of canonical wound healing pathways [18]. Chronic inflammation is commonly associated with infection, aging, diabetes, or idiopathic [19]. TGF-β is a key mediator in regulating fibrotic remodeling, and it promotes activation of fibroblasts, phenoconversion of fibroblasts into highly-contractile, matrix-producing myofibroblasts, and subsequent deposition of extracellular matrix. Previous work has shown that benign prostate stromal fibroblasts will undergo full differentiation into myofibroblast phenotypes when exposed to TGF-β. These cells were shown to express collagens I and III as well as α-SMA [20]. Moreover, chronic inflammation and the presence of proinflammatory cytokines such as interleukin-6 (IL-6), IL-1, IL-8, and IL-15 have been implicated in maintaining fibroblast/myofibroblast activation and promoting extracellular matrix production (Fig. 2) [21].

Preclinical models also support the role of fibrosis as a contributor to the pathogenesis of lower urinary tract dysfunction (LUTD). In young male mice, induction of LUTD with testosterone and estradiol, which recapitulates the hormonal milieu of aging men, leads to increased collagen content within the detrusor wall as well as voiding dysfunction [22]. Furthermore, aged male mice exhibit LUTD that closely phenocopies symptoms experienced by aging men with BPH, and these mice also show increased periurethral and prostate lobe fibrosis [23].

Clinical studies further validate the link between prostate fibrosis and LUTD [13, 14, 24–26]. Post-hoc analysis of prostate needle biopsies from a subset of patients enrolled in the MTOPS trial demonstrated that men who had BPH clinical progression on combination therapy had increased fibrotic changes (increased levels of wavy, aligned collagen) within the prostate transition zone compared to those who did not progress [15]. Similarly, an analysis of routine protocol biopsies taken during the REDUCE trial demonstrated that chronic inflammation was associated with worse IPSS, larger prostate volume, and an increased risk of acute urinary retention [27]. Another study examined 28 consecutive men undergoing radical prostatectomy and studied the mechanical properties and fibrotic changes of periurethral tissue on whole mount specimens [14]. The study found that periurethral prostate tissue from men with worse LUTS (AUA symptom index ≥ 8) was significantly stiffer and had higher collagen content than that from men without LUTS [14].

Taken together, these preclinical and clinical findings support the paradigm in which prostate fibrosis is an important driver of BPH-associated lower urinary tract dysfunction in aging men that is not currently targeted.

## POTENTIAL ANTIFIBROTIC TREATMENTS FOR LOWER URINARY TRACT SYMPTOMS SECONDARY TO BPH

Given the emerging role of fibrosis in the pathogenesis and disease progression of LUTS/BPH, pharmacologic agents with antifibrotic properties show promise as potential novel therapeutic options. Candidate drugs may act through a variety of mechanisms such as inhibition of profibrotic signaling molecules, suppression of proinflammatory mediators, and enhancement of extracellular matrix turnover. Although there are no current FDA-approved antifibrotic medications for the treatment of BPH, preclinical and translational studies provide strong rationale for further investigation.

### Antifibrotic medications approved for fibrotic diseases

Pirfenidone is FDA-approved for the treatment of idiopathic pulmonary fibrosis (IPF) and has been extensively investigated in other fibrotic diseases such as hepatic, renal, and cardiac fibrosis [28–30]. Pirfenidone exerts its antifibrotic effects through reduced TGF-β signaling, inhibition of fibroblast proliferation, and attenuation of profibrotic cytokines and ECM-related genes [29]. In a rat model of chronic prostatitis/chronic pelvic pain syndrome, researchers found that pirfenidone ameliorated chronic pelvic pain (assessed by observing retraction of abdomen, jumping, licking/scratching in response to painful stimuli) and inhibited prostatic inflammation and subsequent fibrosis [31]. Moreover, in a rat model of urethral injury, pirfenidone significantly attenuated the formation of fibrotic urethral strictures compared to untreated animals. Molecular and histopathological analyses showed decreased expression of α-SMA, TGF-β, IL-6, and IL-1β, suggesting that pirfenidone may inhibit both canonical TGF-β signaling and proinflammatory pathways (Fig. 2) [32]. Chronic bladder outlet obstruction secondary to BPH can result in detrusor hypertrophy, fibrosis, and decreased bladder compliance, which may manifest as detrusor underactivity. A recent study showed that treatment of rats with detrusor underactivity with pirfenidone not only improved urinary function but also attenuated fibrotic remodeling of the detrusor wall [33].

Another medication approved for the treatment of IPF is nintedanib, an intracellular tyrosine kinase inhibitor that binds to adenosine triphosphate binding sites and suppresses signaling pathways linked to VEGF, FGF 1–3, and PDGFRα and β; ultimately leading to decreased fibroblast activity (Fig. 2) [34]. Side effects of nintedanib include diarrhea, nausea, and hepatotoxicity [35].

Although the optimal dose for pirfenidone for the treatment of IPF has been established, it is unknown if the same dose is needed for the treatment of prostatic fibrosis and LUTS. Side effects of pirfenidone include skin rash, abdominal pain, decreased appetite, diarrhea, nausea, dizziness, fatigue, headache, upper respiratory tract infection, hepatic dysfunction (elevation of hepatic enzyme levels), and arthralgias [36]. The risk of abnormal liver function (elevated alanine aminotransferase, aspartate aminotransferase, and bilirubin) requires regular monitoring of liver function [37]. Therefore, although treatment is fairly well-tolerated, it is unclear if the potential benefit of pirfenidone/nintedanib for treatment of LUTS/BPH would outweigh the risks. Indeed, if lower dosages of pirfenidone/nintedanib could be used to treat LUTS/BPH, patients might have fewer adverse effects.

### Commonly used urologic medications with antifibrotic potential

Phosphodiesterase-5 inhibitors (PDE5-i) such as sildenafil and tadalafil can be used in the management of LUTS, particularly in patients with comorbid erectile dysfunction, due to their ability to relax smooth muscle tone in the bladder neck and around the prostatic urethra [38]. However, studies have also shown that PDE inhibitors may reduce fibrotic remodeling through cGMP-mediated inhibition of collagen I production and fibroblast to myofibroblast differentiation, and stimulation of fibroblast apoptosis (Fig. 2) [39–41]. Similar evidence can be seen with the drug pentoxifylline, a non-specific cAMP-PDE inhibitor, often used off-label in the treatment of Peyronie’s disease, a condition in which fibrotic plaques develop in the penile tunica albuginea. Studies assessing the effectiveness of PDE inhibition in attenuating fibrosis in a rat model of Peyronie’s disease found that vardenafil and pentoxifylline were both effective in reversing fibrosis (Fig. 2) [40, 42]. Furthermore, in a double-blind randomized controlled trial of 60 patients, combination therapy with pentoxifylline + tamsulosin had superior improvement in IPSS, quality of life, and improvement in Qmax compared to the control group (tamsulosin + placebo) [43]. This suggests that the antifibrotic effect of pentoxifylline augments the therapeutic effect of tamsulosin.

In addition to inhibiting the production of new fibrotic tissue, therapies that break down existing fibrosis may also be effective. Collagenase Clostridium Histolyticum (CCH) is an enzymatic therapeutic for Peyronie’s disease that directly degrades collagen types I and III. While its potential use in the prostate has not been clinically evaluated, targeted enzymatic degradation of dense stromal collagen in the periurethral transition zone could potentially reduce periurethral stiffness and improve voiding function. Indeed, a study assessing the effect of CCH in a rat model of urethral fibrosis found that CCH injections reduced histological fibrosis and expression of collagen types I and III in urethral tissue (Fig. 2) [44]. Side effects of CCH include local injection reactions (pain, hematoma, swelling), erectile dysfunction, and painful erections. Serious side effects included corporal rupture and penile hematoma [45]. Therefore, safety parameters for prostatic CCH injections would need to be clearly defined before using it to treat prostate fibrosis.

### Systemic agents with antifibrotic potential

Thalidomide and its newer analogues (lenalidomide and pomalidomide) are a class of synthetically derived glutamic acid derivatives that have been shown to have robust immunomodulatory and antifibrotic properties [46–49]. Mechanistically, thalidomide exerts its multimodal effects by modulating cereblon, an E3 ubiquitin ligase substrate receptor, leading to downstream degradation of transcription factors involved in canonical inflammatory and profibrotic pathways [50, 51]. Thalidomide has been shown to suppress TGF-β signaling in a variety of fibrotic diseases such as IPF, hepatic cirrhosis, cardiac, renal, and skin fibrosis (Fig. 2) [52–56]. In the context of the lower urinary tract, in vitro studies have demonstrated that thalidomide reduces the proliferation of benign human prostate stromal fibroblasts [57].

Side effects of Thalidomide include: deep venous thrombosis, edema, ischemic heart disease, hyperglycemia, constipation, and peripheral neuropathy [58]. Lenalidomide and pomalidomide are better tolerated than Thalidomide, so they have replaced Thalidomide for the treatment of multiple myeloma [59]. Still, side effects of lenalidomide include: peripheral edema, pruritus, skin rash, diarrhea, nausea, vomiting, anemia, urinary tract infection, dizziness, muscle spasm, and fatigue [60]. Given that thalidomide and its analogues are given systemically and associated with multiple side effects, studies will need to determine the optimal dosing or explore alternative methods of administration that circumvent systemic exposure, e.g., local injection if it is to be used for treatment of LUTS/BPH. Indeed, if lower lenalidomide doses can effectively treat LUTS/BPH, patients may have fewer adverse effects.

### Losartan

Losartan is an Angiotensin II receptor blocker used to treat hypertension and has been shown to have potential antifibrotic activity in the heart, liver, intestine, and renal tissues [61–66]. Angiotensin II has been shown to promote fibrosis through upregulation of TGF-β [64, 67]. In a rat model, unilateral ureteral obstruction (UUO) led to an increase in renal interstitial fibrosis and increased expression of fibrotic proteins such as COL-1, α-SMA, and vimentin compared to control rats [62]. However, administration of Losartan decreased the expression of these fibrotic proteins. The authors then applied transcriptome (RNA) sequencing to screen differentially expressed genes in the three groups (UUO, losartan, and control) to identify potential target genes for the antifibrotic effect of losartan. Ultimately, they identified that administration of losartan decreased mRNA expression of TNF pathway genes, including TNF-α, IL-6, and NF-κβ [62]. In another mouse model of UUO, losartan decreased renal tubular-interstitial injury, fibrosis, and collagen deposition through regulation of the TGF-β/SMAD pathway [63]. Specifically, the authors found that losartan promoted the expression of SMAD7 and E3 ubiquitin-linked enzymes (Smurf 1/2) to degrade the TGF-β1 receptor, ultimately inhibiting renal tubular epithelial cells from undergoing epithelial-mesenchymal transdifferentiation and causing renal fibrosis. In a rat model of neurogenic bladder, transection of spinal nerves led to increased levels of TGF-β1, Type III Collagen, and collagen fibrin expression in bladder tissue [68]. However, administration of losartan decreased expression of these fibrotic markers, prevented loss of bladder compliance, and was protective against the development of hydronephrosis and loss of renal function.

Losartan’s effects have been studied in BPH as well. In a hypertension rat model, those treated with losartan had a significantly decreased prostate to body weight ratio, decreased prostate glandular epithelial content, improved prostatic blood flow, and lower IL-6 and bFGF levels, independent of blood pressure-lowering effects [69]. This suggests that losartan could have anti-inflammatory effects, possibly through regulation of angiotensin II type 1 receptors or restoring prostatic blood flow. The authors also found that high-dose losartan increased apoptosis of glandular epithelial cells in the ventral prostate [69]. Interestingly, another group also identified that angiotensin II type 1 receptors are increased in a human prostate epithelial cell line [70]. Administration of losartan caused a dose-dependent increase in apoptosis in prostate epithelial cells, which was reversed with treatment of an anti-TGF-β antibody. Expression of TGF-β1 was higher in cells treated with losartan, suggesting that blockage of the angiotensin II type 1 receptor caused apoptosis through increased production of TGF-β1 [70].

Mechanistic findings for losartan are not uniform across tissues and models. While antifibrotic effects in renal fibrosis have been associated with attenuation of TGF-β pathways, rat prostate studies with losartan demonstrated increased apoptosis and increased TGF-β, suggesting a context or dose-specific response. Additional work is required to clarify which mechanism is dominant for losartan in the lower urinary tract.

### Veterinary medicines with antifibrotic activity

Halofuginone is a synthetic derivative of febrifugine, a plant alkaloid, that has shown robust antifibrotic potential across a range of organ systems, including the liver, pancreas, esophagus, small intestine, and skin [71, 72]. Its antifibrotic effects have mainly been attributed to inhibition of SMAD3, an immediate secondary messenger in the canonical TGF-β signaling pathway, and direct inhibition of collagen type I expression. Furthermore, halofuginone has also been shown to selectively inhibit differentiation and activation of T-helper 17 (Th17) lymphocytes and downstream T-helper 17 family cytokines such as IL-17A, IL-17F, IL-21, and IL-23, all of which promote fibrotic remodeling and have been implicated in BPH pathogenesis [73–75]. In preclinical urologic animal models, halofuginone has been shown to prevent urethral fibrosis and stricture formation by directly targeting collagen synthesis [76–78]. In a rabbit model of electrocoagulation-induced bulbar urethral stricture, dietary halofuginone significantly reduced the incidence of de novo strictures as well as recurrent strictures following endoscopic urethrotomy [76]. Similar results were seen in rats, in which halofuginone-coated silicone catheters and dietary halofuginone prevented collagen deposition and normalized urethral morphology following periurethral injury [77, 78]. Taken together, these findings highlight the potential for halofuginone as a highly selective antifibrotic agent to target prostate and periurethral fibrosis. Unfortunately, halofuginone is currently only used in veterinary medicine and does not currently have FDA-approved indications for humans. Therefore, the side effect profile of halofuginone in humans is not described.

## IDENTIFICATION OF MEN WITH PROSTATE FIBROSIS

A major barrier to translating antifibrotic therapies into the medical management of LUTS/BPH is phenotyping. Fibrosis is a heterogeneous process, and within the prostate gland, it is further complicated by zone-specific differences in baseline tissue composition. The clinical impact of fibrosis on LUTS depends heavily on collagen superstructure (collagen fiber organization, crosslinking, alignment, and overall stiffness), rather than collagen quantity alone. Prior evidence links periurethral and transition-zone extracellular matrix remodeling with the severity of LUTS and BPH clinical progression; however, this relationship is nuanced and may depend on the spatial distribution of fibrosis relative to the urethra and the resulting mechanical consequences on the tissue [13–15].

Histology remains the gold standard for assessing tissue fibrosis. Common assessments include Masson’s trichrome, picrosirius red, and immunohistochemical analysis of markers of fibroblast activation, myofibroblast differentiation, and collagen subunits. Quantitative or semi-quantitative approaches that allow for analysis of spatial information, such as second-harmonic generation imaging (SHG), have been shown to be increasingly important as they can better relate tissue structure to mechanical behavior and tissue physiology [79]. However, obtaining prostate histology, i.e., prostate biopsy, is not part of routine clinical care for LUTS/BPH, unless there is concurrent PSA elevation or a need to rule out prostate cancer. Thus, noninvasive surrogates to phenotype and quantify prostate fibrosis in vivo are needed if prostate fibrosis is to be a therapeutic strategy in the future. These strategies could also predict patients likely to respond to antifibrotic treatment and assess treatment response.

### Imaging strategies to detect prostate fibrosis

Imaging studies can capture tissue characteristics such as the distribution of prostate fibrosis and periurethral localization in a noninvasive manner. Ultrasound shear-wave elastography (SWE) is a promising imaging technique as it is prostate-accessible (easily adaptable to a transrectal ultrasound workflow) and directly reports stiffness-related parameters. In a cohort of 30 prostate cancer patients, SWE demonstrated measurable differences between malignant and benign tissue, as well as differences in stiffness between transition and peripheral zones [80, 81]. In the context of LUTS/BPH, transrectal SWE has been evaluated specifically in BPH patients in a clinical practice context and showed that quantitative stiffness of the prostate transition zone correlated with age, prostate volume, transition zone volume, and PSA [82]. Magnetic resonance elastography (MRE) is another promising imaging modality to quantify prostate tissue fibrosis, with the advantage of whole gland coverage and integration with standard multiparametric MRI protocols. Initial work established the feasibility of prostate MRE using a specialized transurethral actuator in prostate gelatin phantoms and canine prostate glands, whereas more recent clinical data suggest that high-frequency MRE may be feasible in men imaged for prostatic disease in the setting of LUTS with a transabdominal actuator [83–86]. While additional studies on feasibility are needed, stiffness mapping using ultrasound SWE or MRE shows clear translational potential for identifying BPH patients with increased transition zone stiffness who could respond to antifibrotic therapy or for measuring treatment response.

## OTHER POTENTIAL FUTURE TARGETS TO PREVENT PROSTATE FIBROSIS: ANTI-INFLAMMATORY MEDICATIONS

The ability of immunomodulatory drugs such as thalidomide and lenalidomide to simultaneously target inflammatory and profibrotic pathways may be an advantage in BPH as tissue fibrosis often coexists with chronic inflammation. Indeed, one study identified that patients with a chronic inflammatory infiltrate had a larger prostate volume and were more likely to experience BPH clinical progression and acute urinary retention, compared to those without evidence of inflammation [87]. Furthermore, in another translational study, the authors found that patients with autoimmune disease had a significantly higher incidence of LUTS/BPH [88]. The authors found that macrophage-derived TNF stimulated prostatic fibroblast proliferation, and the blockage of TNF reduced prostatic epithelial hyperplasia. Thus, anti-inflammatory medications could potentially treat LUTS/BPH and prevent prostate fibrosis as well.

## CONCLUSION AND FUTURE DIRECTIONS

Lower urinary tract symptoms are a ubiquitous condition historically attributed to prostatic hyperplasia and smooth muscle dysfunction. These aspects of BPH are effectively targeted by 5-ARIs and α-blockers, respectively. However, some men will progress despite maximum medical treatment with combination therapy, suggesting additional treatment strategies are needed. Indeed, emerging evidence supports that prostate fibrosis contributes to LUTS/BPH. Prostate fibrosis is not currently targeted by any medications for LUTS and, therefore, represents a novel treatment strategy. Mounting evidence supports the clinical use of antifibrotics for treating LUTS. Although antifibrotics such as pirfenidone and lenalidomide are currently FDA-approved for the treatment of other diseases, additional research is needed to determine if antifibrotics can treat LUTS, what the optimal dose or administration route is, and if the medications have acceptable tolerability.
